# Early mobilization in the neurotrauma intensive care unit: a service evaluation of current practice and barriers

**DOI:** 10.3389/fneur.2026.1813263

**Published:** 2026-06-17

**Authors:** Samantha Rooney, James Hodson, Tammy Lea, Hon Sing Geoffrey Wu, Zubair Ahmed, Niharika A. Duggal, Jonathan Weblin, Fiona Howroyd

**Affiliations:** 1 Queen Elizabeth Hospital Birmingham, University Hospitals Birmingham NHS Foundation Trust, Birmingham, United Kingdom; 2 School of Sport, Exercise and Rehabilitation Sciences, University of Birmingham, Birmingham, United Kingdom; 3 Department of Inflammation and Ageing, School of Infection, Inflammation and Immunology, College of Medicine and Health, University of Birmingham, Birmingham, United Kingdom

**Keywords:** barriers, clinical practice, critical care, injury, physiotherapy, rehabilitation

## Abstract

**Background:**

Early mobilization (EM) refers to active exercise commenced while patients are on the intensive care unit (ICU). Although the benefits of EM are well established in general ICU populations, evidence specific to the neurotrauma intensive care unit (NTICU) remains limited. This service evaluation aimed to review current EM practices in a single-center NTICU and explore the perceived barriers to EM implementation.

**Method:**

A service evaluation was conducted from 5th December 2023 to 28th March 2024, including all patients resident in the NTICU. During this period, patients underwent daily physiotherapy assessments, at which data were prospectively recorded for the suitability for EM, and barriers to EM, where this was not performed. Mobility status was quantified using the Manchester Mobility Scale (MMS), with mobilization defined as an MMS ≥ 2 (i.e., sitting on the edge of the bed or higher). Data for patient characteristics and outcomes were retrospectively collected from the electronic health record system.

**Results:**

Of 253 patients included in the service evaluation, EM (i.e., MMS ≥ 2 in the NTICU) was achieved in 149 (58.9%), with a further 76 (30.0%) mobilized on the ward after being discharged from NTICU. The most common primary barriers to EM were sedation (38.4%) and the need for acute medical care (33.9%). Logistical and process issues were the primary barrier to EM in 15.4% of assessments, most commonly insufficient staffing.

**Conclusion:**

This service evaluation is the first known report of perceived barriers to EM in a UK-based NTICU. Targeted strategies to address modifiable barriers may facilitate earlier mobilization within major trauma services. Further research is needed to evaluate the safety and impact of NTICU-specific EM protocols.

## 1 Introduction

The neurotrauma intensive care unit (NTICU) manages patients with severe polytrauma and neurological injuries, such as brain injury, spinal cord injury (SCI), chest trauma, and fractures (1). Survivors often require multiple surgeries, invasive monitoring, and periods of immobility, leading to significant physical and cognitive morbidity and a need for rehabilitation during and after hospital admission (2, 3).

Early mobilization (EM) refers to a range of active interventions commenced while a patient is in the intensive care unit (ICU), which aim to optimize functioning and reduce disability (4–6). Mobilization includes any activity in which patients actively engage their musculature, such as sitting on the edge of the bed or out of bed, standing, or walking, with or without assistance (6, 7). Due to the effects of critical illness and immobility, there may be a need for facilitation from staff. EM aims to prevent muscle wasting, promote neuroplasticity and aid functional recovery following critical illness (8).

National clinical guidelines recommend structured EM for ICU patients, recognizing it as a core component of recovery from critical illness (9, 10). In medical and surgical ICU cohorts, EM is safe, feasible and effective, improving physical and functional outcomes and reducing length of stay (LOS) (11, 12). However, the complexity and heterogeneity of neurotrauma conditions can pose unique challenges for EM delivery, including variability in injury location and severity, and in cognitive status (13, 14). Consequently, patients with neurotrauma are frequently excluded from EM trials in general ICU populations (15). Despite this, emerging evidence supports the feasibility and potential benefits of EM following stroke and acquired brain injury, with improvements reported in mobility, muscle strength and function and quality of life (8, 16). The importance of EM following neurotrauma is further emphasized by longitudinal data indicating that 56% of patients admitted to specialist trauma units require inpatient rehabilitation following hospital discharge (17).

Although there is some encouraging evidence to support EM in neurotrauma populations, there remains a lack of randomized controlled trials within the NTICU to define optimal rehabilitation strategies, including the type, timing, intensity and measurement outcomes in this heterogenous cohort (18). To improve mobilization practices and patient outcomes in the NTICU, it is first necessary to understand current EM practices and the factors that limit their implementation. Therefore, this service evaluation aimed to review EM delivery within a mixed NTICU in a UK major trauma center, and to explore the perceived barriers to EM implementation.

## 2 Methods

### 2.1 Design

This project was registered locally as a service evaluation (CARMS-22399) and reported in accordance with the Strengthening the Reporting of Observational Studies in Epidemiology (STROBE) guidelines (Supplementary File 1) (19).

### 2.2 Setting

This service evaluation was conducted between 5th December 2023 to 28th March 2024 at a regional major trauma center’s NTICU, which admits complex polytrauma and neurosurgical cases and is funded for 27 level three patients. Physiotherapists were staffed at a ratio of 1:5.4 (level three patients), including one clinical specialist, two team leaders, and two senior physiotherapists.

### 2.3 Ethical considerations

This project was deemed to be a service evaluation and not primary research in line with the NHS Health Research Authority; hence, ethical approval was not sought.

### 2.4 Participants

All patients resident in the NTICU with a neurotrauma presentation (based upon clinical diagnosis recorded in medical records) during the period were included in the service evaluation. Patients were excluded if they had opted out of their information being used for research and planning as part of the National Health Service National Data Opt-Out.

### 2.5 Variables and data measurement

A physiotherapist visited all patients daily on weekdays and assessed their suitability to engage in EM activity; appropriate EM or in-bed rehabilitation was then performed. EM was not performed on weekends, public holidays, or days of insufficient staffing due to sickness or leave, during which only emergency respiratory physiotherapy was provided where required. Although an EM protocol existed, this was not specific to neurotrauma populations; hence, decisions around EM suitability and practice were based on individual clinical judgment.

#### 2.5.1 Prospective daily assessment of mobilization activity and barriers

At each daily assessment in the NTICU, physiotherapists prospectively collected data on treatment-related factors that may have impacted the ability of patients and therapists to engage in active EM activities, including the use of mechanical ventilation, sedation, inotropes, haemofiltration, intracranial pressure bolts (ICP) and cerebral spinal fluid (CSF) diversion devices. All factors for safely undertaking EM were considered from pre-existing evidence and recommendation for safe EM in the ICU (7). The level of consciousness was assessed using the Glasgow Coma Scale (GCS) for non-sedated patients, or the Richmond Agitation Sedation Scale (RASS) for sedated patients (20, 21). Mobility status was quantified at each physiotherapy session using the Manchester Mobility Scale (MMS; Table 1) (22).

**Table 1:** Manchester mobility scale (MMS).

MMS score	Definition
0	Unable to complete/agitated/refused/too unwell
1	Passive exercises in bed
2	Sitting on the edge of the bed
3	Passive transfer to a chair
4	Standing practice
5	Step transfer to chair with assistance
6	Mobilizing less than 30 m
7	Mobilizing more than 30 m

The MMS definition is based on (22), m = metres.

Where mobilization activity did not occur, the primary perceived barrier was recorded by the treating physiotherapist from a list of 17 options, with an additional option to provide a free-text response where none of these were applicable. For analysis, the 17 barriers were collated into five categories: sedation, acute medical care (including medical/haemodynamic, respiratory or neurological instability), logistics/processes (including removal of lines, staffing, or a delayed mobility plan), injury management (including spinal precautions or trauma/plastics restrictions to mobilization and weight bearing, or upcoming surgery), and patient related factors (including agitation, patient declining input, or limited by pain). The data collection tool was devised by a senior physiotherapist and consultant physiotherapist specializing in ICU rehabilitation (Supplementary File 2). A short trial period was undertaken, and education was provided prior to all physiotherapy staff collecting data. Any uncertainty was discussed with a senior physiotherapist.

#### 2.5.2 Retrospective review of NTICU admission

Additional patient data were retrospectively extracted from the electronic health record (EHR) system, including age, gender, diagnosis at NTICU admission; the timing and duration of sedation, ventilation and tracheostomies; the overall LOS in NTICU and hospital; and the discharge destination. Where patients had multiple NTICU stays during the same hospital admission, the timing of the assessments were defined based on the date of the initial NTICU admission, and the NTICU LOS was calculated as a total across all NTICU stays. All durations were calculated based on calendar days, such that the day of NTICU admission was treated as day zero.

The prospectively collected assessment data was then retrospectively reviewed to identify the date of first mobilization. This was defined as the first assessment with MMS ≥ 2 (i.e., sitting on the edge of the bed or higher) (6). Where this did not occur in the NTICU, but still within the acute hospital setting, the EHR was reviewed to identify the date that this occurred on the subsequent hospital ward.

For patients with at least one physiotherapy contact where EM was not performed due to a modifiable barrier (i.e., logistics and processes), a retrospective review of the EHR was performed. Two senior ICU physiotherapists (who were not directly involved in the patient’s clinical care) reviewed the EHR, to determine the earliest timepoint at which EM could potentially have been achieved in the absence of modifiable barriers. Documented modifiable barriers were identified through structured interrogation of physiotherapy, medical and nursing entries. To ensure that the modifiable barrier was the sole impediment to mobilization, all other potential barriers to EM (as defined in Supplementary File 2) were cross-checked for the same time period. The potential time to EM was calculated as the difference (in days) between the date of actual first mobilization and the earliest date of which mobilization could reasonably have occurred, had modifiable barriers not been present. This assessment was based on documented physiological stability, clinical status and mobilization readiness, as recorded in the EHR and represents a conservative estimate of potentially avoidable delay in EM. Any uncertainty in cases was resolved by discussion between the two physiotherapists, until consensus was achieved.

### 2.6 Statistical methods

All analyses were performed using IBM SPSS v29 (IBM Corp. Armonk, NY), with *p* < 0.05 deemed to be indicative of statistical significance throughout. Continuous variables were summarized as “mean ± standard deviation” where approximately normally distributed, or as “median (interquartile range; IQR)” otherwise. The timing of the first mobilization (MMS ≥ 2) was assessed using a Kaplan–Meier curve approach, with patients censored at death or hospital discharge. For patients who mobilized in hospital, characteristics were compared between those first mobilized in NTICU vs. post-NTICU using Mann–Whitney *U* tests for ordinal or continuous variables, or Fisher’s exact tests for nominal variables. Where mobilization was not performed, changes over time in the distribution of barriers to mobilization were analyzed using a Kruskal–Wallis test on the assessment-level data (i.e., by comparing the average time from NTICU admission to assessment between the barriers).

## 3 Results

### 3.1 Cohort characteristics

A total of 253 neurotrauma patients were resident in the NTICU during the study period, all of whom had at least one assessment by a physiotherapist. The cohort had a mean age of 52 ± 18 years and 62.8% were male. Patients most commonly presented with trauma (37.5%) or acquired brain injuries (36.4%). Sedation was required during the NTICU stay in 70.8% of patients and mechanical ventilation in 71.1%. The median hospital LOS was 22 days (IQR: 10–43), of which a median of 6 days (IQR: 3–14) were in the NTICU, with 3.6% of patients having multiple admissions to NTICU during their hospital stay. A total of 23 (9.1%) patients died in NTICU, and a further 10 (4.0%) patients died in hospital after being discharged from NTICU. Intrahospital transfers were performed in 43 (17.0%) patients, with one (0.4%) patient still being in hospital at the time of data collection (6 months from NTICU admission). No ongoing rehabilitation after hospital discharge was required for 22.5% of patients, with 23.3% requiring home rehabilitation, while either neurological or general inpatient rehabilitation was required in 18.6 and 5.1%, respectively. Further details of the cohort are reported in Tables 2, 3.

**Table 2:** Cohort characteristics.

Characteristic	*N*	Statistic
Age (years)	253	52 ± 18
Male gender	253	159 (62.8%)
Primary diagnosis	253	
Trauma		95 (37.5%)
Acquired brain injury		92 (36.4%)
Neuro-oncological		20 (7.9%)
Other neurological		34 (13.4%)
Spinal surgery		12 (4.7%)
Traumatic brain injury	253	53 (20.9%)
Severity of TBI^a^	52	
Mild (GCS: 13–15)		10 (19.2%)
Moderate (GCS: 9–12)		9 (17.3%)
Severe (GCS: 3–8)		33 (63.5%)
Spinal injury	253	28 (11.1%)
Pelvic injury	253	15 (5.9%)
Chest trauma	253	32 (12.6%)
Long bone fractures	253	29 (11.5%)
CSF diversion device	253	47 (18.6%)
Intracranial pressure bolt	253	55 (21.7%)
Sedation	253	179 (70.8%)
Duration (days)^b^	179	4 (2–9)
Mechanical ventilation	253	180 (71.1%)
Duration (days)^b^	180	6 (2–14)
Tracheostomy	253	51 (20.2%)
NTICU admission to placement (days)^b^	51	10 (8–14)

Analyses are on the patient-level data, and are based on *N* = 253, unless stated otherwise. Data are reported as *N* (%), median (interquartile range), or as mean ± standard deviation, as appropriate. ^a^Based on the GCS at the scene of the TBI, in patients with TBIs where data were available. ^b^In patients where the stated treatment was performed. CSF, cerebrospinal fluid; GCS, Glasgow Coma Scale; NTICU, neurotrauma intensive care unit; TBI, traumatic brain injury.

**Table 3:** Patient outcomes.

Outcomes	*N*	Statistic
Mobilized in hospital	253	
No		28 (11.1%)
In NTICU		149 (58.9%)
On ward (post-NTICU discharge)		76 (30.0%)
MMS at first mobilization in NTICU ^a^	149	
2		75 (50.3%)
3		4 (2.7%)
4		16 (10.7%)
5		35 (23.5%)
6		7 (4.7%)
7		12 (8.1%)
NTICU LOS (days)^b^	253	6 (3–14)
Multiple NTICU admissions	253	9 (3.6%)
Hospital LOS (days)^bc^	253	22 (10–43)
Hospital discharge destination	253	
Died in hospital		33 (13.0%)
Inpatient neuro. rehab.		47 (18.6%)
Inpatient general rehab.		13 (5.1%)
Home – with rehab.		59 (23.3%)
Home – no rehab.		57 (22.5%)
Still in hospital		1 (0.4%)
Intrahospital transfer		43 (17.0%)

Analyses are on the patient-level data, and are based on *N* = 253, unless stated otherwise. Data are reported as *N* (%), median (interquartile range), or as mean ± standard deviation, as appropriate. ^a^In patients who mobilized in NTICU. ^b^Defined as the time from admission to discharge, death or transfer. ^c^One patient was still in hospital; hence the date of data collection was used to calculate the LOS. NTICU, neurotrauma intensive care unit; MMS, Manchester Mobility Score; LOS, length of stay; Rehab., rehabilitation.

### 3.2 Mobilization status

**Figure 1:**
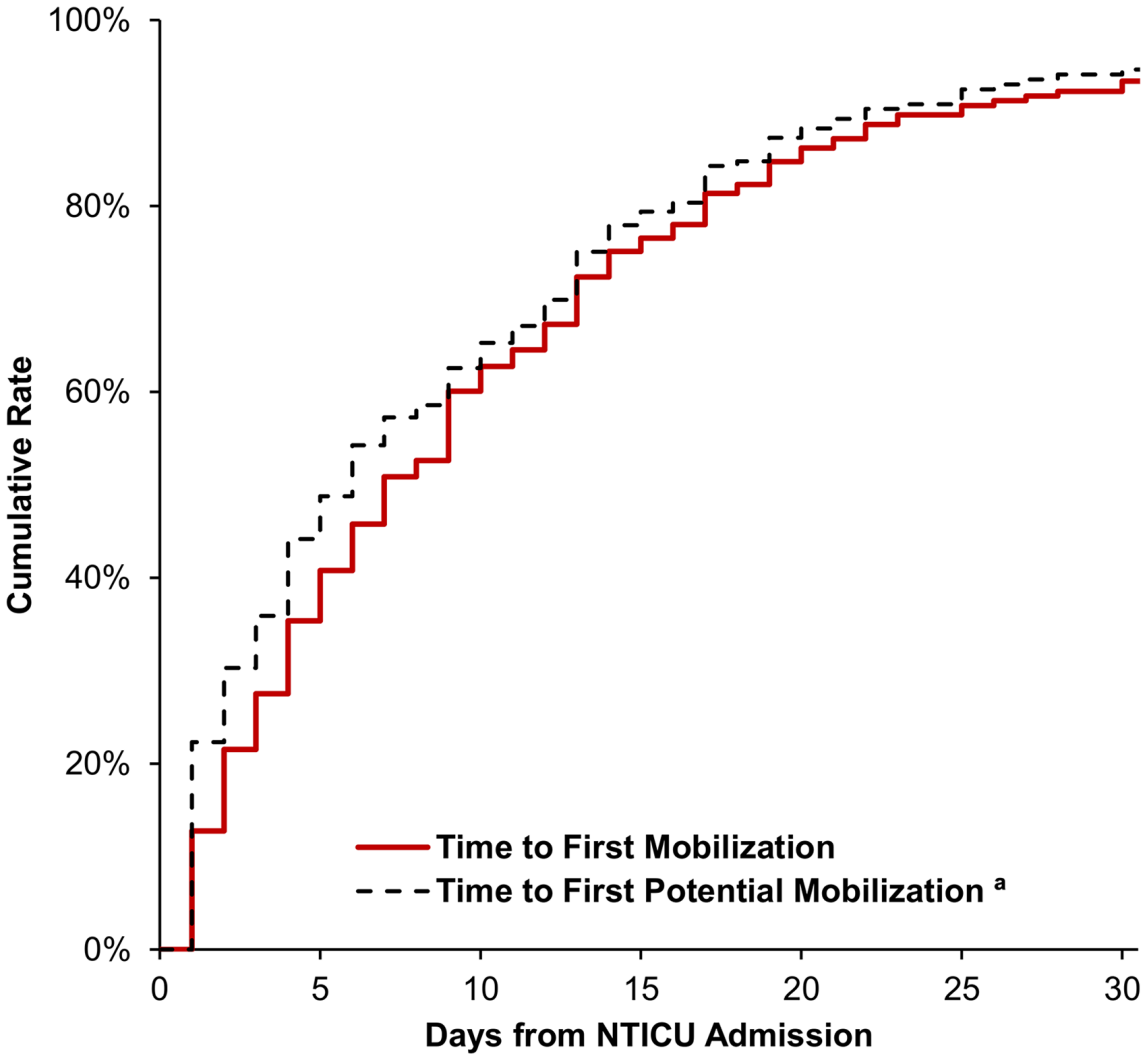
Kaplan–Meier curve of time to first mobilization. Patients are censored at death or hospital transfer. The *x*-axis is truncated at 30 days. ^a^Represents the time to the earliest potential mobilization if the “logistics/processes” barriers were removed. NTICU: neurotrauma intensive care unit.

The EM was achieved in 149 (58.9%) patients during the NTICU admission, with a further 76 (30.0%) mobilized on the ward post-NTICU discharge. Mobilization was not achieved during inpatient hospitalization in the remaining 28 (11.0%), due to mortality (*N* = 27) or intrahospital transfer (*N* = 1). Kaplan–Meier analysis returned a median time to mobilization of 7 days (IQR: 2–13), with cumulative mobilization rates of 12.7, 50.9, 75.1 and 87.2% after 1, 7, 14 and 21 days, respectively (Figure 1). In patients who achieved mobilization in hospital, demographic- and injury-related factors were similar in those where this first occurred in NTICU vs. on a hospital ward post-NTICU discharge (Table 4). However, significant differences in treatment-related factors were observed, with patients first mobilized in NTICU being more likely to require ICP bolts, sedation, mechanical ventilation or tracheostomy, while those mobilized post-NTICU were more likely to have CSF diversion devices. A significant difference in the NTICU LOS was also observed, with a median of 7 days (IQR: 4–18) for those first mobilized in NTICU compared to 4 days (IQR: 2–9) for those mobilized post-NTICU (*p* < 0.001).

**Table 4:** Cohort characteristics by location of first mobilization.

Cohort characteristics	*N*	Location of first mobilization		*p*-value
		In NTICU	Post-NTICU	
Age (years)	225	51 ± 18	50 ± 18	0.452
Male gender	225	97 (65.1%)	44 (57.9%)	0.310
Primary diagnosis	225			0.355
Trauma		58 (38.9%)	28 (36.8%)	
Acquired brain injury		53 (35.6%)	23 (30.3%)	
Neuro-oncological		9 (6.0%)	11 (14.5%)	
Other neurological		21 (14.1%)	10 (13.2%)	
Spinal surgery		8 (5.4%)	4 (5.3%)	
Traumatic brain injury	225	34 (22.8%)	13 (17.1%)	0.387
Severity of TBI^a^	46			0.246^a^
Mild (GCS: 13–15)		9 (27.3%)	1 (7.7%)	
Moderate (GCS: 9–12)		6 (18.2%)	3 (23.1%)	
Severe (GCS: 3–8)		18 (54.5%)	9 (69.2%)	
Spinal injury	225	11 (7.4%)	11 (14.5%)	0.101
Pelvic injury	225	8 (5.4%)	5 (6.6%)	0.766
Chest trauma	225	19 (12.8%)	11 (14.5%)	0.836
Long bone fractures	225	18 (12.1%)	9 (11.8%)	1.000
CSF diversion device	225	15 (10.1%)	25 (32.9%)	**<0.001**
Intracranial pressure bolt	225	39 (26.2%)	10 (13.2%)	**0.027**
Sedation	225	109 (73.2%)	43 (56.6%)	**0.016**
Duration (days)^b^	152	5 (2–10)	3 (2–7)	**0.026**
Mechanical ventilation	225	109 (73.2%)	43 (56.6%)	**0.016**
Duration (days)^b^	152	9 (3–17)	4 (2–9)	**0.003**
Tracheostomy	225	46 (30.9%)	4 (5.3%)	**<0.001**
NTICU admission to placement (days)^b^	50	10 (8–14)	11 (5–15)	0.706
NTICU LOS (days)^c^	225	7 (4–18)	4 (2–9)	**<0.001**
Multiple NTICU admissions	225	4 (2.7%)	4 (5.3%)	0.448
Hospital LOS (days)^cd^	225	22 (11–42)	29 (15–49)	0.059

Analyses are on the patient-level data for the *N* = 225 patients who were mobilized in hospital (defined as MMS ≥2), unless stated otherwise. Continuous variables are reported as median (interquartile range), or as mean ± standard deviation, as appropriate, with *p*-values from Mann–Whitney *U* tests. Categorical variables are reported as *N* (%), with *p*-values from Fisher’s exact tests, unless stated otherwise. Bold *p*-values are significant at *p* < 0.05. ^a^Based on the GCS at the scene of the TBI, in patients with TBIs where data were available; the *p*-value is from a Mann–Whitney *U* test, as the factor is ordinal. ^b^In patients where the stated treatment was performed. ^c^Defined as the time from admission to discharge, death or transfer. ^d^One patient was still in hospital; hence the date of data collection was used to calculate the LOS. CSF, cerebrospinal fluid; GCS, Glasgow Coma Scale; LOS, length of stay; NTICU, neurotrauma intensive care unit; TBI, traumatic brain injury.

### 3.3 Clinical therapies and EM rates

**Figure 2:**
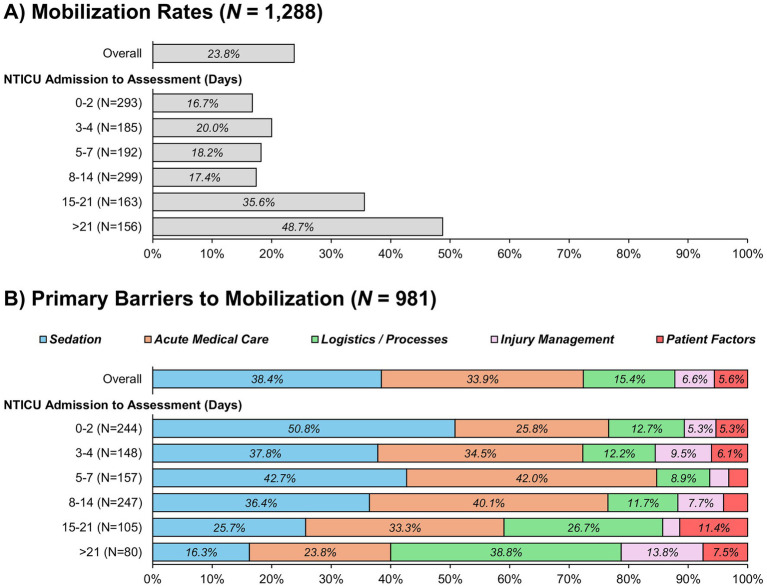
Mobilization rates and primary barriers by day since NTICU admission. **(A)** Mobilization rates by days in the NTICU. **(B)** Primary barriers to mobilization during NTICU admission. Analyses were performed on the assessment-level data. Initially, the overall mobilization rate, and the distribution of primary barriers to mobilization for assessments where this was not performed were calculated. In addition to reporting overall rates, assessments were grouped based on the time from NTICU admission to assessment. Unlabeled bars comprise <5% of cases. NTICU, neurotrauma intensive care unit.

The prospective audit was performed on a total of 78 working days, during which time a total of 1,288 assessments were completed, with a median of 18 assessments per day (IQR: 10–21) and 3 per patient (IQR: 2–7, maximum: 36). Thirty-five days were not included in the service evaluation, due to being weekends or public holidays, with a further 2 days excluded due to insufficient staffing levels resulting from sickness absence and annual leave. Patient characteristics and outcomes at the time of each assessment are summarized in Table 5. EM was achieved in a total of 23.8% (*N* = 307) of assessments, with rates increasing from 16.7% of assessments performed between day 0–2, to 48.7% of those performed after >21 days of patients’ NTICU stay (Figure 2A).

**Table 5:** Clinical therapies and mobilization rates at daily assessments.

Daily assessment data	*N*	Statistic
Airway	1,288	
Endotracheal tube		523 (40.6%)
Tracheostomy		287 (22.3%)
Spontaneous ventilation		478 (37.1%)
Mechanical ventilation	1,288	652 (50.6%)
Sedation	1,288	384 (29.8%)
Inotropes	1,288	293 (22.7%)
Haemofiltration	1,288	17 (1.3%)
Intracranial pressure bolt	1,288	86 (6.7%)
CSF diversion device	1,288	206 (16.0%)
GCS^a^	904	
13–15		370 (40.9%)
9–12		307 (34.0%)
3–8		227 (25.1%)
RASS^a^	384	
−5		103 (26.8%)
−4		166 (43.2%)
−3		64 (16.7%)
−2		38 (9.9%)
−1		13 (3.4%)
Mobilized	1,288	307 (23.8%)
MMS	1,288	
0		124 (9.6%)
1		857 (66.5%)
2		115 (8.9%)
3		64 (5.0%)
4		31 (2.4%)
5		59 (4.6%)
6		12 (0.9%)
7		26 (2.0%)

Analyses are on the assessment-level data, and are based on *N* = 1,288, unless stated otherwise. All variables were recorded as of the time of the assessment. ^a^GCS is reported for patients who were not sedated at the time of the assessment, with RASS used for those that were sedated. CSF, cerebrospinal fluid; GCS, Glasgow Coma Scale; MMS, Manchester Mobility Score.

### 3.4 Perceived barriers to EM

For the 981 assessments where EM was not performed, the most common perceived primary barriers were sedation (38.4%) and the need for acute medical care (33.9%). Logistical and process issues were the primary barrier to EM in 15.4% of assessments, most commonly insufficient staffing. For the remainder, the primary barriers were injury management (6.6%) or patient factors (5.6%) (Table 6). The distribution of these barriers changed significantly over patients’ NTICU stay (*p* < 0.001, Figure 2B). Specifically, the proportion of assessments where sedation was the primary barrier to EM decreased from 50.8 to 16.3% for assessments performed between 0–2 days and >21 days after NTICU admission, with a corresponding increase from 12.7 to 38.8% in assessments where logistical and process issues were the primary barrier.

**Table 6:** Primary perceived barriers to early mobilization.

Barrier	*N* (%)
Sedation	**377 (38.4%)**
Acute medical care	**333 (33.9%)**
Medical instability	*86 (8.8%)*
Neurological instability	*85 (8.7%)*
GCS/ETT	*65 (6.6%)*
EVD	*46 (4.7%)*
Respiratory instability	*35 (3.6%)*
Medical/surgical procedure	*16 (1.6%)*
Logistics/processes	**151 (15.4%)**
Staffing	*110 (11.2%)*
Delayed mobility plan	*16 (1.6%)*
Nurse-led	*12 (1.2%)*
Awaiting specialist seating	*10 (1.0%)*
Removal of Line	*3 (0.3%)*
Injury management	**65 (6.6%)**
Spinal precautions	*53 (5.4%)*
T&O/plastics restrictions	*12 (1.2%)*
Patient factors	**55 (5.6%)**
Agitation	*39 (4.0%)*
Patient declined	*11 (1.1%)*
Pain	*5 (0.5%)*

Analyses are on the assessment-level data and include the *N* = 981 assessments at which mobilization activity did not occur. The perceived barriers are those recorded by the treating physiotherapist, using the data collection tool in Supplementary File 2. Rates are primarily reported for broader categories (bold), which are subsequently broken down into sub-categories (italic) – all percentages are based on a denominator of *N* = 981. ETT, endotracheal tube; EVD, external ventricular drainage; GCS, Glasgow Coma Scale; T&O, trauma and orthopaedic.

Since logistical and process barriers were potentially modifiable by improvements to staffing levels and processes, the potential impact of such changes was then assessed. A review of patients with at least one assessment with logistical and process issues as the primary barrier to EM (*N* = 95) identified 50 (19.8% of overall cohort) patients who could potentially have been mobilized earlier, by a median of 4 days (IQR: 2–7), had this barrier been removed. Consequently, addressing logistical and process barriers could potentially have reduced the Kaplan–Meier estimated median time to mobilize for the cohort to 6 days (IQR: 1–12), equivalent to an average improvement of 1 day (Figure 1).

## 4 Discussion

This service evaluation examined current EM practices in a single-center NTICU and explored perceived barriers to EM implementation. Over a four-month period, 253 patients and 1,288 physiotherapy contacts were analyzed. Most physiotherapy contacts comprised bed-based activity (i.e., MMS 0–1; 76.2%), with only 58.9% of patients achieving EM (i.e., MMS ≥ 2) at least once during their NTICU admission. Mobilization rates increased with time from admission, from 16.7% during contacts on days 0–2 to 48.7% for contacts occurring after 21 days. The most commonly identified barriers to EM were sedation, acute medical needs, and logistical issues. Among patients with potentially modifiable barriers, alleviating staffing and process barriers may have enabled 19.8% of patients to mobilize earlier, by a median of 4 days, reducing the median time to mobilize for the cohort as a whole by 1 day. Despite an in-hospital survival rate of 87.0%, patient outcomes remained poor, with a median hospital stay of 22 days, 47.0% requiring ongoing rehabilitation, and 17.0% transferred for ongoing care. Collectively, these findings highlight the clinical importance of optimizing rehabilitation delivery within the NTICU, and the need for prospective studies to improve patient outcomes.

Although national guidelines recommend EM as a core component of critical care recovery, only 58.9% of patients in this cohort were mobilized during their NTICU admission. This is substantially lower than previously reported mobilization rates from our institution, which reported that 100% of patients admitted with COVID-19 and requiring mechanical ventilation were mobilized during their ICU stay (23, 24). Similarly, the TEAMS study reported a mobilization rate of 89.2% among patients who required mechanical ventilation (25). Notably, these studies excluded patients with significant neurotrauma. The NTICU treats a breadth of pathologies which often necessitate multi-specialty input which can limit EM (26). In our cohort, over one third of patients (37.5%) sustained traumatic injuries, including spinal injuries (11.1%), pelvic injuries (5.9%) and long bone fractures (11.5%), which may necessitate periods of immobilization to facilitate surgical fixation or protect healing structures. However, there is ongoing debate about the optimal surgical timing to minimize complications and enable earlier mobilization (27). Additionally, 18.6% of patients required CSF diversion devices, which have traditionally necessitated bed-rest precautions. Despite recent evidence supporting the safety and feasibility of mobilization with CSF diversion devices, significant variation in practice persists across centers in Europe (28–30). Although the heterogeneity in our cohort may suggest potential barriers to EM, the association between injury subgroups and EM has not been explored in this service evaluation and, thus, the effects are only speculative. Despite our diverse and critically ill cohort, our analysis indicates that EM is achievable in some patients within the NTICU. However, further research is required to establish the safety and efficacy of EM in specific neurotrauma populations.

Although 58.9% of patients were mobilized at least once during their NTICU stay, only 23.8% of physiotherapy sessions involved mobilization (MMS ≥ 2), consistent with previously reported challenges of EM in the NTICU (31). This service evaluation included patients with GCS < 15 and complex polytrauma, which may explain why just 3.0% of daily interventions achieved an MMS ≥ 6 (i.e., mobilizing any distance). Patients with reduced GCS may lack cognitive capacity to participate in consistent and safe rehabilitation; features that are common after brain injury (14). In our cohort, 63.5% of TBI cases were deemed severe (GCS: 3–8), and 21.7% of patients required ICP monitoring. Shock resolution, ICP instability, and perfusion management also pose significant medical challenges after acute brain injury, due to the risk of secondary brain injury, which may further limit the ability to engage in active EM in the NTICU (14). Additionally, the requirement of sedation in 70.8% of patients for a median of 4 days and ventilation in 71.1% for a median of 6 days may also reflect illness severity during the acute stages of admission, when the safety of out-of-bed mobilization may be limited. Nonetheless, the frequency of EM improved over the duration of patients’ NTICU stay, from 16.7% of interventions on days 0–2 compared to 48.7% after 21 days. This potentially demonstrates an acute period of medical instability following NTICU admission, and that recovery can be non-linear and protracted, regardless of injury severity (32).

The second aim of this service evaluation was to identify perceived barriers to EM. Sedation (38.4%) and acute medical care (33.9%) were the most frequent reported barriers overall; however, these shifted over time toward potentially modifiable logistical and process-related factors. Beyond 21 days, logistical issues, most commonly staffing, became the dominant barrier. This finding is supported by the comparison between patients mobilized in the NTICU and those mobilized following transfer to ward environments, with mobilization in the NTICU more commonly occurring in the context of sedation, ventilation and ICP monitoring. In contrast, patients mobilized post-NTICU were more likely to have CSF diversion devices, where mobilization may be delayed by staffing constraints or uncertainty regarding management of the drain, particularly in the absence of established standard operating procedures. Notably, in patients who achieved EM, over a third achieved MMS ≥ 5 at their first mobilization (36.2%), suggesting a subgroup with the capacity to progress to weight bearing activity and ambulation, should barriers to EM be alleviated. Identifying patients at greatest risk of immobilization and long-term rehabilitation needs could help direct future research and targeted interventions in an under-resourced health system, particularly in the NTICU where caseloads are complex and heterogenous.

Providing patients with earlier and structured rehabilitation has the potential to improve patient functional outcomes, reduce the duration of mechanical ventilation and LOS and, therefore, reduce health-care related costs (15, 33). In our service evaluation, among patients with staffing and process barriers to EM, just under 20% could potentially have mobilized earlier, by a median of 4 days, reducing the median time to mobilize for the cohort as a whole by 1 day. Given that a single ICU bed-day in the UK costs in excess of £1,500, addressing modifiable barriers to EM represents a potential opportunity for both clinical and economic benefits (34). Improving mobility status at ICU discharge also has the potential to reduce hospital LOS and increase the likelihood of discharge to usual residence (35). With nearly half of patients in this cohort required ongoing rehabilitation and a substantial proportion required onward acute transfers, these findings reinforce the urgent need to optimize rehabilitation delivery within the NTICU.

### 4.1 Strengths and limitations

This service evaluation had several strengths, including the large sample size and detailed prospective data collection. However, there are also limitations which need to be considered when interpreting the results. Primarily, the single-center service evaluation design means that the results may not be generalizable to other centers. Due to the nature of our NTICU (situated within a Major Trauma Centre, specialist neurosurgical center and hosting the Royal Centre for Defence Medicine), there may be differences in the complexity of the patient population, treatment protocols or multidisciplinary staffing ratios and expertise, potentially limiting the generalisability to other centers. Furthermore, our current physiotherapy staffing model does not provide a weekend EM service, whereas other centers may utilize a seven-day rehabilitation service. These factors are particularly pertinent to in the NTICU, which comprises a heterogenous patient group and there is a lack of guidance on the optimum approach to EM, potentially leading to large variability between centers. Secondly, there will have been some subjectivity when identifying the primary perceived barrier to EM, particularly in cases where multiple barriers could potentially apply. While perceived barriers were standardized, there may have been variation in the levels of clinical expertise and confidence across the individual physiotherapists undertaking the daily assessments, which may have influenced results. However, this was minimized through staff training and opportunity to discuss with uncertainty with a senior physiotherapist, in addition to a preliminary pilot of the data collection tool. Thirdly, assessments were not performed on 37 days of the analysis period, due to it being a weekend, public holiday or due to staffing issues. As such, the results are only applicable to the current staffing model and may not be generalizable to a 365-day service. Finally, data were not available for markers of patients’ injury severity (e.g., the APACHE II score), or for whether any adverse events occurred during the EM sessions. Future studies should therefore consider clinical decision-making and safety of EM practices in the NTICU, in addition to patient-focused outcomes on the effectiveness of EM upon functional recovery.

## 5 Conclusion

This service evaluation is the first known report of perceived barriers to EM in a UK NTICU. Although 58.9% of patients were mobilized (MMS ≥ 2) during their NTICU admission, mobilization occurred in fewer than one quarter of physiotherapy sessions, with very low rates of active ambulation. Survivors had prolonged hospital stays and high rehabilitation needs, likely reflecting neurological severity and clinical complexity. In the acute phase, sedation and intensive medical care were key barriers, along with intrinsic functional limitations. Logistical and processes-related factors, particularly staffing, were identified as modifiable barriers to EM, and addressing these could meaningfully reduce time to mobilization in affected patients. Despite cohort heterogeneity, early mobilization appears feasible in neurotrauma patients. However, further research is urgently required to evaluate the safety, feasibility and effectiveness of NTICU-specific EM protocols, and to determine their impact on both short- and long-term functional outcomes, with a view to improving patient care and outcomes following admission to the NTICU.

## Data Availability

De-identified data from this study are not available in a public archive. De-identified data from this study will be considered to be made available upon direct request to the corresponding authors. Requests to access these datasets should be directed to Fiona Howroyd, fiona.howroyd@uhb.nhs.uk; Jonathan Weblin, jonathan.weblin@uhb.nhs.uk.
