# Supplementary material for: Early mobilization in the neurotrauma intensive care unit: a service evaluation of current practice and barriers
Source: Front Neurol. 2026 Jun 17;17:1813263. doi: 10.3389/fneur.2026.1813263 (PMC13318685; doi:10.3389/fneur.2026.1813263)
Supplement: Supplementary file 1 [file Data_Sheet_1.PDF]

Supplementary Material

Supplementary File 1 – STROBE Checklist

STROBE Statement—Checklist of items that should be included in reports of ***cohort studies***

	Item No	Recommendation	Page No
**Title and abstract**	1	(*a*) Indicate the study's design with a commonly used term in the title or the abstract	Title page
		(*b*) Provide in the abstract an informative and balanced summary of what was done and what was found	Abstract
**Introduction**			
Background/rationale	2	Explain the scientific background and rationale for the investigation being reported	Section 1. Introduction
Objectives	3	State specific objectives, including any prespecified hypotheses	Section 1. Introduction
**Methods**			
Study design	4	Present key elements of study design early in the paper	Section 2.1 Setting & design
Setting	5	Describe the setting, locations, and relevant dates, including periods of recruitment, exposure, follow-up, and data collection	Section 2.1 Setting & design
Participants	6	(*a*) Give the eligibility criteria, and the sources and methods of selection of participants. Describe methods of follow-up	Section 2.1 Setting & design
		(*b*) For matched studies, give matching criteria and number of exposed and unexposed	N/A
Variables	7	Clearly define all outcomes, exposures, predictors, potential confounders, and effect modifiers. Give diagnostic criteria, if applicable	Section 2.2 Data collection
Data sources/ measurement	8*	For each variable of interest, give sources of data and details of methods of assessment (measurement). Describe comparability of assessment methods if there is more than one group	Section 2.2 Data collection & Supp. File 1
Bias	9	Describe any efforts to address potential sources of bias	Section 2.2 Data collection &

			2.3 Statistical methods
Study size	10	Explain how the study size was arrived at	N/A
Quantitative variables	11	Explain how quantitative variables were handled in the analyses. If applicable, describe which groupings were chosen and why	Section 2.3 Statistical methods
Statistical methods	12	(*a*) Describe all statistical methods, including those used to control for confounding	Section 2.3 Statistical methods
		(*b*) Describe any methods used to examine subgroups and interactions	
		(*c*) Explain how missing data were addressed	
		(*d*) If applicable, explain how loss to follow-up was addressed	
		(*e*) Describe any sensitivity analyses	
**Results**			
Participants	13*	(a) Report numbers of individuals at each stage of study—eg numbers potentially eligible, examined for eligibility, confirmed eligible, included in the study, completing follow-up, and analysed	Section 3.1 Cohort Characteristics
		(b) Give reasons for non-participation at each stage	
		(c) Consider use of a flow diagram	
Descriptive data	14*	(a) Give characteristics of study participants (eg demographic, clinical, social) and information on exposures and potential confounders	Section 3.1 Cohort Characteristics and Table 2a
		(b) Indicate number of participants with missing data for each variable of interest	
		(c) Summarise follow-up time (eg, average and total amount)	
Outcome data	15*	Report numbers of outcome events or summary measures over time	Throughout section 3

Main results	16	(*a*) Give unadjusted estimates and, if applicable, confounder-adjusted estimates and their precision (eg, 95% confidence interval). Make clear which confounders were adjusted for and why they were included	N/A
		(*b*) Report category boundaries when continuous variables were categorized	
		(*c*) If relevant, consider translating estimates of relative risk into absolute risk for a meaningful time period	
Other analyses	17	Report other analyses done—eg analyses of subgroups and interactions, and sensitivity analyses	Throughout section 3
**Discussion**			
Key results	18	Summarise key results with reference to study objectives	Section 4
Limitations	19	Discuss limitations of the study, taking into account sources of potential bias or imprecision. Discuss both direction and magnitude of any potential bias	Section 4.1

Interpretation	20	Give a cautious overall interpretation of results considering objectives, limitations, multiplicity of analyses, results from similar studies, and other relevant evidence	Section 5. Conclusions
Generalisability	21	Discuss the generalisability (external validity) of the study results	Section 4. Discussion
**Other information**			
Funding	22	Give the source of funding and the role of the funders for the present study and, if applicable, for the original study on which the present article is based	Funding section

*Give information separately for exposed and unexposed groups.

Note: An Explanation and Elaboration article discusses each checklist item and gives methodological background and published examples of transparent reporting. The STROBE checklist is best used in conjunction with this article (freely available on the Web sites of PLoS Medicine at http://www.plosmedicine.org/, Annals of Internal Medicine at http://www.annals.org/, and Epidemiology at http://www.epidem.com/). Information on the STROBE Initiative is available at http://www.strobe-statement.org.

Supplementary Material

Supplementary File 2 – Data Collection Tool

**Patient ID:**	………………	**Date:**	………………	
**Airway:**	☐ ETT	☐ Trache.	☐ NIV	☐SV
**Mechanical Ventilation:**	☐ No	☐ Yes		
**Sedation:**	☐ No	☐ Yes		
**Inotropes / Vasopressors:**	☐ No	☐ Yes		
**Hemofiltration (CVVH):**	☐ No	☐ Yes: Neck	☐ Yes: Groin	
**ICP Bolt:**	☐ No	☐ Yes		
**EVD / Lumbar Drain:**	☐ No	☐ Yes		
**Spinal Precautions:**	☐ No	☐ Yes		
**GCS *(if Non-Sedated)***	***E:*** ………	***V:*** ………	***M:*** ………	
**RASS Score *(if Sedated)*:**	………			
**MMS Score:**	………			

Primary Reason Not Mobilised *(if MMS<2 - Please Select One Option)*:

☐ Sedated	☐ Pain	☐ Agitation
☐ Patient Declined	☐ Medical Instability	☐ Neuro. Instability
☐ Resp. Instability	☐ T&O/Plastics Restrictions	☐ Spinal Precautions
☐ GCS / ETT (M<6)	☐ Medical/Surgical Procedure	☐ EVD
☐ Removal of Line	☐ A/w Specialist Seating	☐ Delayed Mobility Plan
☐ Nurse-Led Mobilisation	☐ Staffing	

☐ Other (Please State) …………………………………………………………………………..
